# Infant Formula Supplemented with Five Human Milk Oligosaccharides Shifts the Fecal Microbiome of Formula-Fed Infants Closer to That of Breastfed Infants

**DOI:** 10.3390/nu15143087

**Published:** 2023-07-10

**Authors:** Andrea Q. Holst, Pernille Myers, Paula Rodríguez-García, Gerben D. A. Hermes, Cathrine Melsaether, Adam Baker, Stina R. Jensen, Katja Parschat

**Affiliations:** 1 Chr. Hansen A/S, 2970 Hoersholm, Denmark; dkgerh@chr-hansen.com (G.D.A.H.); dkcmm@chr-hansen.com (C.M.); dkadb@chr-hansen.com (A.B.); dkstaj@chr-hansen.com (S.R.J.); 2 Clinical Microbiomics, 2200 Copenhagen, Denmark; pernille@clinical-microbiomics.com (P.M.); paula@clinical-microbiomics.com (P.R.-G.); 3 Chr. Hansen HMO GmbH, 53619 Rheinbreitbach, Germany; dekapa@chr-hansen.com

**Keywords:** human milk oligosaccharides, breastfeeding, infant formula, gut microbiome development, bifidobacteria, infant-type bifidobacteria, gut metabolic modules, aromatic lactic acids

## Abstract

Breastmilk is the optimal source of infant nutrition, with short-term and long-term health benefits. Some of these benefits are mediated by human milk oligosaccharides (HMOs), a unique group of carbohydrates representing the third most abundant solid component of human milk. We performed the first clinical study on infant formula supplemented with five different HMOs (5HMO-mix), comprising 2′-fucosyllactose, 3-fucosyllactose, lacto-*N*-tetraose, 3′-sialyllactose and 6′-sialyllactose at a natural total concentration of 5.75 g/L, and here report the analysis of the infant fecal microbiome. We found an increase in the relative abundance of bifidobacteria in the 5HMO-mix cohort compared with the formula-fed control, specifically affecting bifidobacteria that can produce aromatic lactic acids. 5HMO-mix influenced the microbial composition as early as Week 1, and the observed changes persisted to at least Week 16, including a relative decrease in species with opportunistic pathogenic strains down to the level observed in breastfed infants during the first 4 weeks. We further analyzed the functional potential of the microbiome and observed features shared between 5HMO-mix-supplemented and breastfed infants, such as a relative enrichment in mucus and tyrosine degradation, with the latter possibly being linked to the aromatic lactic acids. The 5HMO-mix supplement, therefore, shifts the infant fecal microbiome closer to that of breastfed infants.

## 1. Introduction

Human breastmilk provides the optimal nutritional composition and contains bioactive compounds that meet the requirements of infants. Exclusive breastfeeding is, therefore, recommended from birth to 6 months of age [1]. After lactose and lipids, human milk oligosaccharides (HMOs) are the third most abundant solid components of breastmilk [2]. HMOs are complex carbohydrates that are indigestible by humans but contribute to healthy development in early life [3]. These carbohydrates are recognized as prebiotics, promoting the growth of beneficial bacteria in the gut [4], but also act independently by directly interacting with intestinal epithelial cells to support gut barrier function [5], promoting immune system maturation [6] and conferring protection against pathogenic bacteria and viruses by acting as decoy receptors [5,7,8,9,10]. HMOs are composed of a lactose backbone that is elongated via different linkages with d-glucose, d-galactose, *N*-acetylglucosamine, l-fucose and *N*-acetylneuraminic acid (sialic acid). These combinations result in more than 200 structurally distinct HMOs and can be categorized into three types: neutral non-fucosylated, neutral fucosylated, and acidic or sialylated [2,11]. The HMO profile is unique for each mother and changes during lactation. However, the 10 most abundant HMOs account for more than 70% of the total HMO concentration on average, with highly variable individual concentrations due to genetic, environmental and geographic factors [12,13].

Infants are born with a naïve gastrointestinal tract, and the first months of life are characterized by a fluctuating microbiome influenced by birth mode and exposure to environmental bacteria [14]. The consumption of breastmilk is the most influential factor in the composition of the early-life gut microbiome [15,16]. Breastmilk promotes the colonization of particular bifidobacterial species via the prebiotic effect of HMOs [17], and bifidobacteria associated with breastfeeding can promote a healthy immune system by producing aromatic lactic acids [18]. However, only 44% of infants born today are exclusively breastfed during the first 6 months—most are supplemented with or exclusively fed infant formula [1]. Infant formula products have been developed to match the nutritional composition of human breastmilk but lack many bioactive components, such as HMOs, which were unavailable as a formula ingredient until 2016 [19].

Recent technological advances have enabled the inclusion of 2′-fucosyllactose (2′FL), the most abundant HMO naturally, in infant formulas. Clinical studies have shown that formulas supplemented with 2′FL are safe and well-tolerated by infants [20,21,22,23], and other studies have confirmed the safety of combinations of two HMOs [6] and specific blends of five HMOs at low [24] and high concentrations [25,26]. Interestingly, the microbiome of formula-fed infants becomes more similar to that of breastfed infants when HMOs are included [23,24]. HMO supplements also reduce the relative abundance of specific bacterial pathogens and are linked to fewer incidents of respiratory tract infections when compared with infants fed control formula [6,27,28]. Further infant health benefits may arise when more complex HMO mixes are included in formula products at ratios and concentrations closer to those found in human breastmilk [25,26]. We previously showed that infants fed formula supplemented with 5HMO-mix—a blend of 2’FL, 3-fucosyllactose (3FL), lacto-*N*-tetraose (LNT), 3′-sialyllactose (3’SL) and 6′-sialyllactose (6’SL) at a total concentration of 5.75 g/L, developed to simulate both the concentration and diversity of HMOs in human milk [29]—was safe and well-tolerated [26]. Here, we describe microbiome development during the first 6 months after birth in a cohort of infants fed on breastmilk (BM) compared with infants fed formula supplemented with 5HMO-mix and infants fed control formula (IF control).

## 2. Materials and Methods

### 2.1. Cohort Characteristics

Clinical data and samples representing 311 infants were collected from 12 sites across Germany, Italy and Spain between December 2018 and November 2020. The primary aim of the study was to assess tolerability and safety, and details of study design, procedures and clinical outcomes are reported elsewhere [26]. Eligible subjects were healthy female and male infants ≤14 days of age at visit 1 (V1), born at full term (37–42 weeks of gestational age), singleton birth, with a birth weight of 2500–4500 g and an APGAR score of 9 or 10 as assessed within the first 15 min after birth. Infants were excluded from the study if they had (a) any congenital disorders that could affect the study outcome or subject safety, (b) self-reported parental or sibling allergy to cow’s milk protein or (c) administration of antibiotics prior to V1 and concurrent treatment with any medicine or natural health products unless medically indicated by a professional healthcare provider. In addition, subjects were excluded if parents were not prepared to feed the subject solely within the assigned study group. Moreover, the adverse events incidence rate was reported equivalent in the three feeding groups.

The study followed the principles of the Declaration of Helsinki and good clinical practice. All procedures involving human subjects were approved by the relevant ethics committees for each site. Written informed consent was obtained from all parents or legal guardians of the subjects before any study-related procedures were initiated. The study was registered at clinicaltrials.gov (ID NCT03513744) before enrolling the first subject. The clinical baseline characteristics of the infants are shown in Table S1.

The infant formula provided to the subjects in the 5HMO-mix group and the formula control group was equal in quantities of proteins, lipids, vitamins and other nutrients, and the final concentration of 5.75 g/L 5HMO-mix was achieved by partially replacing the carbohydrates (maltodextrin) in the basic formula with 5HMO-mix powder (4.35 g/100 g formula powder) [26].

### 2.2. Sample Collection

Infant fecal samples were obtained in Week 1, Week 2, Week 4, Week 8, Week 12 and Week 16, and 6 months after enrolment in the clinical study (Table 1). The fecal samples were voluntarily collected by the parents. The first sample was collected after visit 1, while the following fecal samples were collected the week before the next visit. The microbiome study was not designed with a baseline because the infants received the respective formula before the first sampling. Due to corona restrictions at some sites, remote visits were initiated, and the fecal samples could not be obtained. In the breastfed group, only two samples were obtained at 6 months (BM, N = 2). Microbiome analysis was the secondary endpoint of the clinical study with which the safety, tolerability and effects on infant growth were evaluated as the primary endpoints.

### 2.3. Fecal DNA Extraction and Sequencing

Microbial DNA was extracted from ~0.1 g aliquots of fecal samples using a NucleoSpin 96 Soil kit (Macherey-Nagel) with horizontal bead beating in a Vortex-Genie 2 at 2700 rpm for 5 min. The extracted DNA was randomly sheared into ~350 bp fragments, and a library was constructed using the NEBNext Ultra Library Prep Kit for Illumina (New England Biolabs). The DNA libraries were evaluated using a Qubit 2.0 fluorimeter and an Agilent 2100 Bioanalyzer to determine the fragment size distribution, and with quantitative real-time PCR to determine the concentration. We carried out 2 × 150 bp paired-end sequencing using the Illumina platform. Raw sequencing data were filtered to remove host contamination by discarding read pairs that mapped to human reference genome GRCh38 with Bowtie2 v2.4.2 [30]. After trimming to remove adapters and bases with a Phred score below 20 using AdapterRemoval v2.3.1 [31], read pairs ≥100 bp were retained as high-quality non-host reads.

### 2.4. Microbiome Taxonomic Annotation and Profiling

Microbial taxonomic annotation was achieved using the metagenomics species (MGS) concept [32]. High-quality non-host reads were mapped to the Clinical Microbiomics in-house Human Gut gene catalog (containing 14,355,839 genes) using BWA mem v0.7.17 [33]. An individual read was considered uniquely mapped to a gene if it aligned with ≥95% identity over ≥100 bp and the mapping quality (MAPQ) was ≥20. Reads meeting the alignment identity and length criteria but not the MAPQ threshold were considered to be multi-mapped. Reads with >10 nonaligned bases or bases extending beyond the gene were considered unmapped. Read pairs were classified into one of three possible categories: unmapped (read pairs in which both individual reads were unmapped), multi-mapped (read pairs in which both individual reads were multi-mapped or one was multi-mapped and the other was unmapped) and mapped to a gene (read pairs in which both individual reads mapped to the same gene, or in which one read mapped to a gene and the other was unmapped or multi-mapped, or mapped to another gene in the same MGS). A gene count matrix was created with the number of uniquely mapped read pairs for each gene.

Taxonomic abundance profiling was carried out using the Clinical Microbiomics in-house set of Human Gut Metagenomics Species (2095 MGS), where each MGS is represented by a set of 100 signature genes from the gene catalog, optimized for accurate abundance profiling. An MGS count matrix was created based on the total signature gene counts from each MGS. However, an MGS was considered to be detected only if reads were uniquely mapped to at least three signature genes. The MGS count matrix was normalized according to effective gene length and normalized sample-wise to relative abundance estimates for each MGS.

MGS were taxonomically annotated by using their genes as BLAST queries against the NCBI RefSeq prokaryotic genomes (19 January 2022) and nt (3 August 2021) databases and by applying discrete annotation criteria for subspecies, species, genus, family, order, class, phylum and superkingdom. On this basis, a taxon was assigned to an MGS if at least 75%, 75%, 60%, 50%, 40%, 30%, 25% and 20% of the MGS genes were mapped to the taxon if no more than 10%, 10%, 10%, 20%, 20%, 20%, 20% and 15%, respectively, of the given MGS genes were mapped to a different taxon and if the BLAST hits featured alignment length ≥100 bp; query coverage ≥50%; and identity ≥95%, 95%, 85%, 75%, 65%, 55%, 50% and 45%, respectively. Each MGS was processed with CheckM [34], and its annotation was updated if the CheckM outcome resulted in a lower taxonomic rank.

### 2.5. Microbiome Functional Annotation and Profiling

Potential functional profiles were determined using Gut Metabolic Modules (GMMs), a set of 103 conserved metabolic pathways defined as a series of enzymatic steps represented by Kyoto Encyclopedia of Genes and Genomes (KEGG) orthology (KO) identifiers [35]. KO annotations were obtained by mapping the gene catalog to the EggNOG v5.0 orthologous groups database using EggNOG-mapper v2.0.1 in Diamond mode [36]. GMMs were assigned to the MGS if all KO annotations (for modules with fewer than four steps) or at least two-thirds of the KO annotations from any reaction path (modules with at least four steps) were present in the given MGS.

Additional potential functional modules were manually annotated, one for *Bifidobacterium*-specific fucose degradation and two for sialic acid degradation. The *Bifidobacterium*-specific fucose degradation module was annotated based on the pathway found in *Bifidobacterium longum* subsp. *infantis* (KO identifiers: K02431, K07046, K18334, K22397 and K02429 [37]). The two sialic acid degradation modules were annotated based on the pathway found in *Escherichia coli* (KO identifiers: K01639, K00885, K01877, K01443 and K02564 [38]) and the pathway found in *Bifidobacterium breve* (KO identifiers: K01714, K02564, K25026, K01788 and K01443 [39]). These modules were assigned to the MGS if at least three-fifths of the KO annotations in a module were present in the given MGS.

*Bifidobacterium* species were functionally categorized based on their ability to produce aromatic lactic acids using an aromatic lactate dehydrogenase (ALDH). *Bifidobacterium* strains containing ALDH included *B. longum* subsp. *longum*, *B. longum* subsp. *infantis*, *B. bifidum*, *B. breve* and *B. scardovii* [18].

### 2.6. Microbiome Ecological Measures and Statistical Analysis

Microbiome analysis was carried out using R v4.1.1. Microbiome diversity was measured by calculating alpha and beta diversity on a down-sampled MGS abundance matrix generated by random sampling read counts without replacement. Alpha diversity was calculated as the richness and Shannon index (using the R package vegan). Beta diversity was calculated as the weighted UniFrac distance (using the R package phyloseq). Beta diversity was compared among the feeding groups with permutational multivariate analysis of variance (PERMANOVA) using the feeding group as explanatory variable and compared between infant genders in the 5HMO-mix group with PERMANOVA using gender as explanatory variable. The PERMANOVA test was carried out using the adonis2 function in the R package vegan with 1000 permutations and with = “margin”.

Pairwise statistical comparisons among feeding groups at different taxonomic levels (MGS, species, genus, family, order, class and phylum) and in different functional modules were performed using the Mann–Whitney U-test. Effect sizes were calculated using Cliff’s delta statistic. When testing multiple hypotheses, the Benjamini–Hochberg method was used to ensure that the false discovery rate (FDR) remained ≤10%.

### 2.7. Machine Learning Predictions of Clinical Variables

A proprietary machine learning pipeline based on the R package mlr3 [40] was trained to predict clinical outcomes using the microbiome composition (MGS level) with a random forest (RF) algorithm. The clinical outcomes of interest included tolerability endpoints (defined as stool frequency and consistency), digestive tolerance parameters (regurgitation, vomiting and flatulence) and behavioral parameters (fussiness, crying and awakening at night). Performance (root mean squared error (RMSE), mean absolute error (MAE) and R-squared (R^2^)) was evaluated using five-fold cross-validation, while hyperparameters were tuned within each fold using holdout resampling with a random search tuning algorithm. To counter the bias of having multiple samples from the same individual, cross-validation was blocked by subject to ensure that the model was never trained and tested on samples from the same individual [41]. Birth mode, feeding group, gestational age, smoking in the household and antibiotic use were included as confounders in the model.

## 3. Results

### 3.1. The Fecal Microbial Composition Differs among Feeding Groups

**Figure 1:**
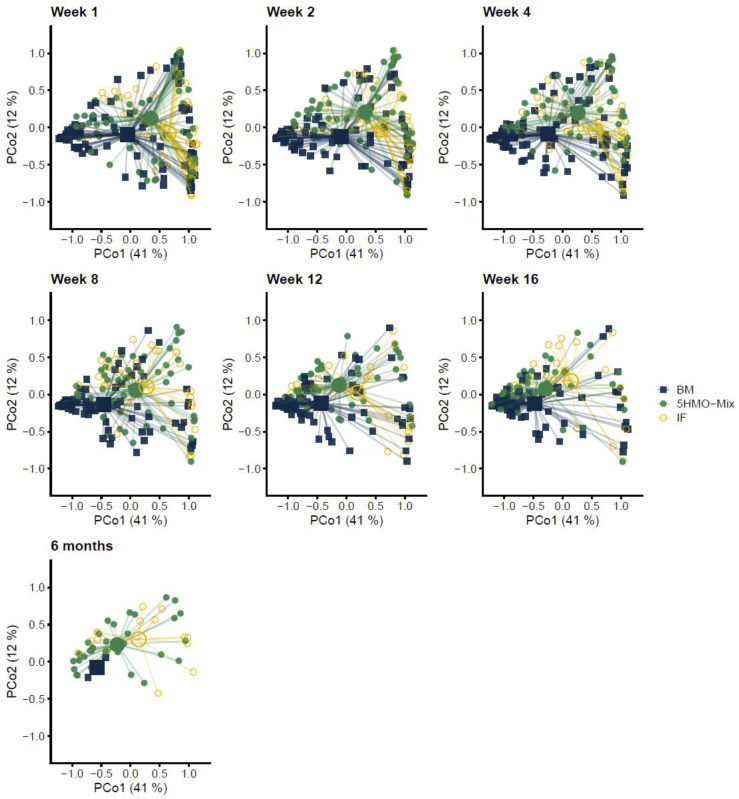
Infant fecal microbial composition visualized using principal coordinates analysis (PCoA) based on weighted UniFrac distances. Feeding groups are color-coded, and the plots, faceted by time point. Individual data points are shown by the centroid of each group being indicated by a larger shape. The axis labels indicate the microbial variance explained by the first two principal coordinates. Significance (*p*-values) and effect sizes (*R*^2^) for permutational multivariate analysis of variance (PERMANOVA) using feeding group as explanatory variable for pairwise comparisons among groups in Weeks 1–16, and between 5HMO-mix and IF at 6 months are shown in Table S2. BM = breastmilk; 5HMO-mix; IF = control infant formula. Numbers in each cohort (N) are provided in Table 1.

The association between feeding groups and fecal microbial composition was first assessed using weighted UniFrac distances. Principal coordinates analysis (PCoA) revealed significant separation of the three feeding groups across the first two PC axes from Week 1 to Week 16 (R^2^ > 8.5%, *p* < 0.001) (Figure 1 and Table S2). Differences were already observed in Week 1 when assuming an immediate effect of the presence of HMOs in infant formula. Breastfed and formula-fed infants differed significantly in microbial composition at all time points (R^2^ > 6.5%, *p* < 0.012). This suggests that breastfeeding is the main driver explaining the difference in microbial composition. Comparing the breastfed group with each formula group showed that the IF control differed at all time points (R^2^ > 9.5%, *p* < 0.001), whereas the 5HMO-mix group differed to a greater extent in Weeks 2–8 (R^2^ > 7.3%, *p* < 0.001) than Week 1 (R^2^ = 4.7%, *p* < 0.001), Week 12 (R^2^ = 5.6%, *p* < 0.003) and Week 16 (R^2^ = 3.3%, *p* < 0.012). Moreover, the 5HMO-mix and IF control groups differed significantly at all time points (R^2^ > 3%, *p* < 0.03), except Week 8 (R^2^ = 1.7%, *p* > 0.1). These data show that the 5HMO-mix supplement influenced the microbial composition, which became more similar to that of breastfed infants. This influence was not different between 5HMO-mix male and female infants at any of the time points (R^2^ < 5%, *p* > 0.1). In general, we found no indication that microbiome composition could be used to predict the clinical outcomes in this study (Table S3).

### 3.2. The Relative Abundance of Bifidobacteria Increases in the 5HMO-Mix Feeding Group

**Figure 2:**
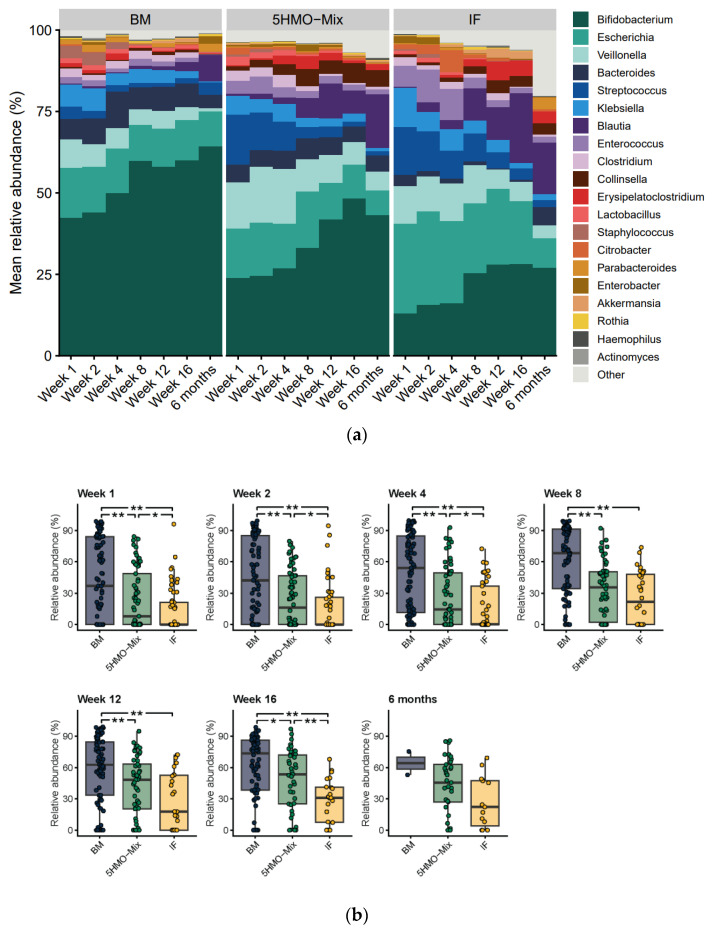
Profile of the infant fecal microbiome and the relative abundance of *Bifidobacterium* according to feeding group. (**a**) Relative abundance of the 20 most abundant genera (average > 85% of the analyzed MGS) averaged within each feeding group at each time point. (**b**) Relative abundance of fecal bifidobacteria in each feeding group at each time point. Boxplots show medians as horizontal lines; box boundaries indicate the interquartile range; and whiskers represent values within 1.5× the interquartile range of the first and third quartiles. MGS are identified at the genus level. Significance of pairwise comparisons was calculated using the Mann–Whitney U test (* *p* < 0.05, ** *p* < 0.01). BM = breastmilk; 5HMO-mix; IF = control infant formula. Numbers in each cohort (N) are provided in Table 1.

The relative abundance of fecal bacteria was quantified using the MGS concept. The 20 most abundant genera (on average > 80% of MGS) across the three feeding groups included *Bifidobacterium*, *Escherichia*, *Veillonella*, *Bacteroides*, *Streptococcus*, *Klebsiella*, *Blautia* and *Enterococcus* (Figure 2a). The most significant differences were observed when comparing breastfed and formula-fed infants. The relative abundance of *Bifidobacterium* was significantly higher in the breastfed group at all time points compared with the IF control (*p* < 0.001) and 5HMO-mix (*p* < 0.02) groups (Figure 2b), whereas the relative abundance of genera including *Escherichia*, *Streptococcus*, *Klebsiella*, *Blautia* and *Enterococcus* was significantly lower (Figure S1a,d–g). When comparing the most abundant genera, the 5HMO-mix group showed trends similar to those of the breastfed group relative to the IF control, with higher abundance of *Bifidobacterium* in Weeks 1–4 (*p* < 0.01) and 16 (*p* < 0.005) (Figure 2b), whereas the relative abundance of *Escherichia* and *Enterococcus* was significantly lower (Figure S1a,g). The relative abundance of the genus *Veillonella* was significantly higher in the 5HMO-mix group than in the breastfed group in Weeks 1–16 (*p* < 0.02) (Figure S1b), whereas the genus *Bacteroides* was significantly more abundant compared with IF control infants in Week 1 and Weeks 8–12 (*p* < 0.05) (Figure S1c). Large individual variations among infants were, however, observed within the groups, with the relative abundance of *Bifidobacterium* ranging from >90% to <1%.

### 3.3. The 5HMO-Mix Supports Bifidobacterium Species That Are Able to Produce Aromatic Lactic Acids

**Figure 3:**
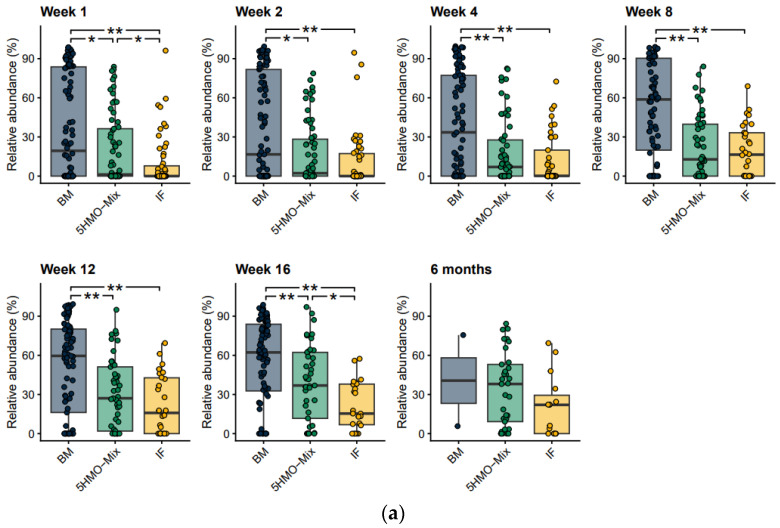

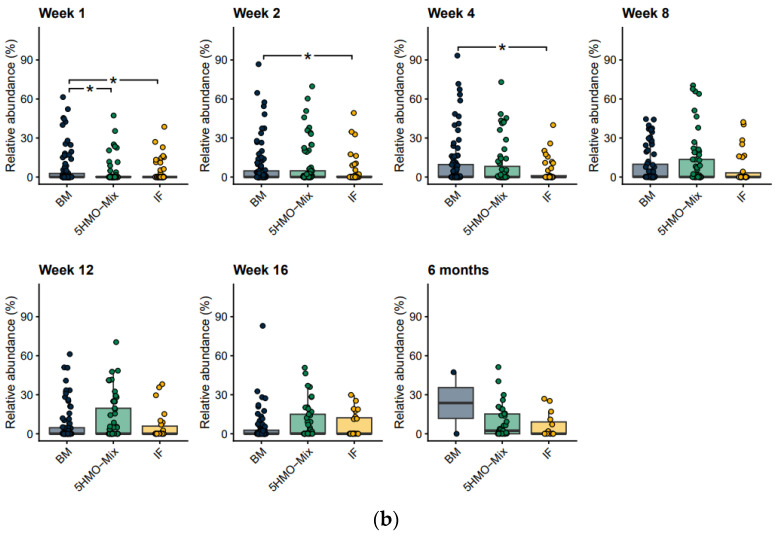
Relative abundance of *Bifidobacterium* MGS able to produce aromatic lactic acids. (**a**) *Bifidobacterium* MGS with (ALDH^+^) and (**b**) without (ALDH^–^) are shown on boxplots at each time point and are compared among feeding groups. The ability to produce aromatic lactic acids is derived directly from the relative abundance of MGS that carry the ALDH genes. Boxplots show medians as horizontal lines; box boundaries indicate the interquartile range; and whiskers represent values within 1.5× the interquartile range of the first and third quartiles. Significance in pairwise comparisons was calculated using the Mann–Whitney U test (* *p* < 0.05, ** *p* < 0.01). BM = breastmilk; 5HMO-mix; IF = control infant formula. Numbers in each cohort (N) are provided in Table 1.

*Bifidobacterium* species associated with breastfeeding can be characterized by the presence of an aromatic lactate dehydrogenase (ALDH^+^) giving them the ability to produce aromatic lactic acids, a metabolite shown to interact with immune cells and affect cytokine production [18]. We found that ALDH^+^ *Bifidobacterium* was more abundant than ALDH^−^ *Bifidobacterium* in the breastfed group with a similar trend in the 5HMO-mix group, with significant differences from the IF control group in Week 1 and Week 16 (*p* < 0.05) (Figure 3a,b). This may indicate that 5HMO-mix specifically promotes the growth of *Bifidobacterium* species associated with breastfeeding. The most abundant (on average across groups and time points > 10% MGS) *Bifidobacterium* species were *B. longum* subsp. *longum*, *B. bifidum*, *B. breve*, *B. adolescentis*, *B. catenulatum* subsp. *kashiwanohense*, *B. dentium*, *B. longum* subsp. *infantis* and *B. pseudocatenulatum* (Figure S2a–h). Breastfed infants showed a higher relative abundance of the ALDH^+^ species *B. bifidum* and *B. breve* than the two formula-fed groups (Figure S2a,b), whereas the relative abundance of *B. longum* subsp. *longum* was similar in the breastfed and 5HMO-mix groups at all time points (Figure S2d). The remaining ALDH^+^ species *B. longum* subsp. *infantis* (Figure S2c) and *B. scardovii* were characterized by a low prevalence and low abundance across the three groups.

### 3.4. The Relative Abundance of Opportunistic Pathogens Is Reduced by 5HMO-Mix

**Figure 4:**
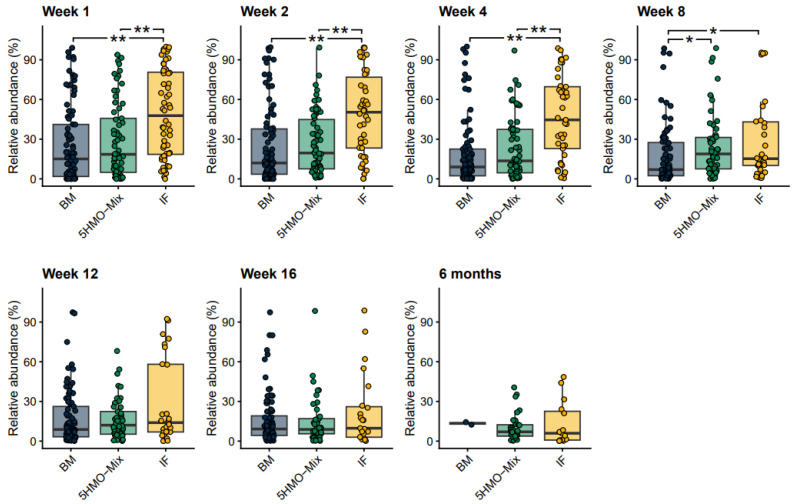
Relative abundance of opportunistic pathogen MGS summed for each time point and compared among feeding groups. Opportunistic pathogen MGS were identified based on the species name matched to a predefined list [42]. Boxplots show medians as horizontal lines; box boundaries indicate the interquartile range; and whiskers represent values within 1.5× the interquartile range of the first and third quartiles. Significance in pairwise comparisons was calculated using the Mann–Whitney U test (* *p* < 0.05, ** *p* < 0.01). BM = breastmilk; 5HMO-mix; IF = control infant formula. Numbers in each cohort (N) are provided in Table 1.

Commensal gut bacteria include some of the most prevalent opportunistic pathogens, such as strains of *Escherichia coli*, *Enterococcus faecalis*, *Clostridioides difficile*, *Klebsiella pneumoniae* and *Streptococcus agalactiae*. A predefined list of 33 bacterial species associated with the global burden of bacterial infections was summed at each time point within each feeding group [42]. We found that the relative abundance of these species was lower in the breastfed and 5HMO-mix groups than in the IF control group (Figure 4). Interestingly, the difference was more significant at earlier time points (Weeks 1–4, *p* < 0.01) and non-significant after Week 12 (*p* > 0.05). Compared with the IF control group, breastfed infants had significantly lower relative abundance of *E. coli* in Week 2, Week 8 and Week 12 (*p* < 0.05) and of *C. difficile* in Weeks 4–16 (*p* < 0.05). Considering the same species, the only significant difference between the breastfed and 5HMO-mix groups was *E. coli* in Week 8 (*p* < 0.05) (Figure S3a,b). *K. pneumoniae* was significantly less abundant in the breastfed group than in both formula groups at all time points (*p* < 0.02), except for the 5HMO-mix group in Week 16 (*p* > 0.05) (Figure S3c).

### 3.5. 5HMO-Mix Directs the Functional Potential of the Microbiome towards That of Breastfed Infants

**Figure 5:**
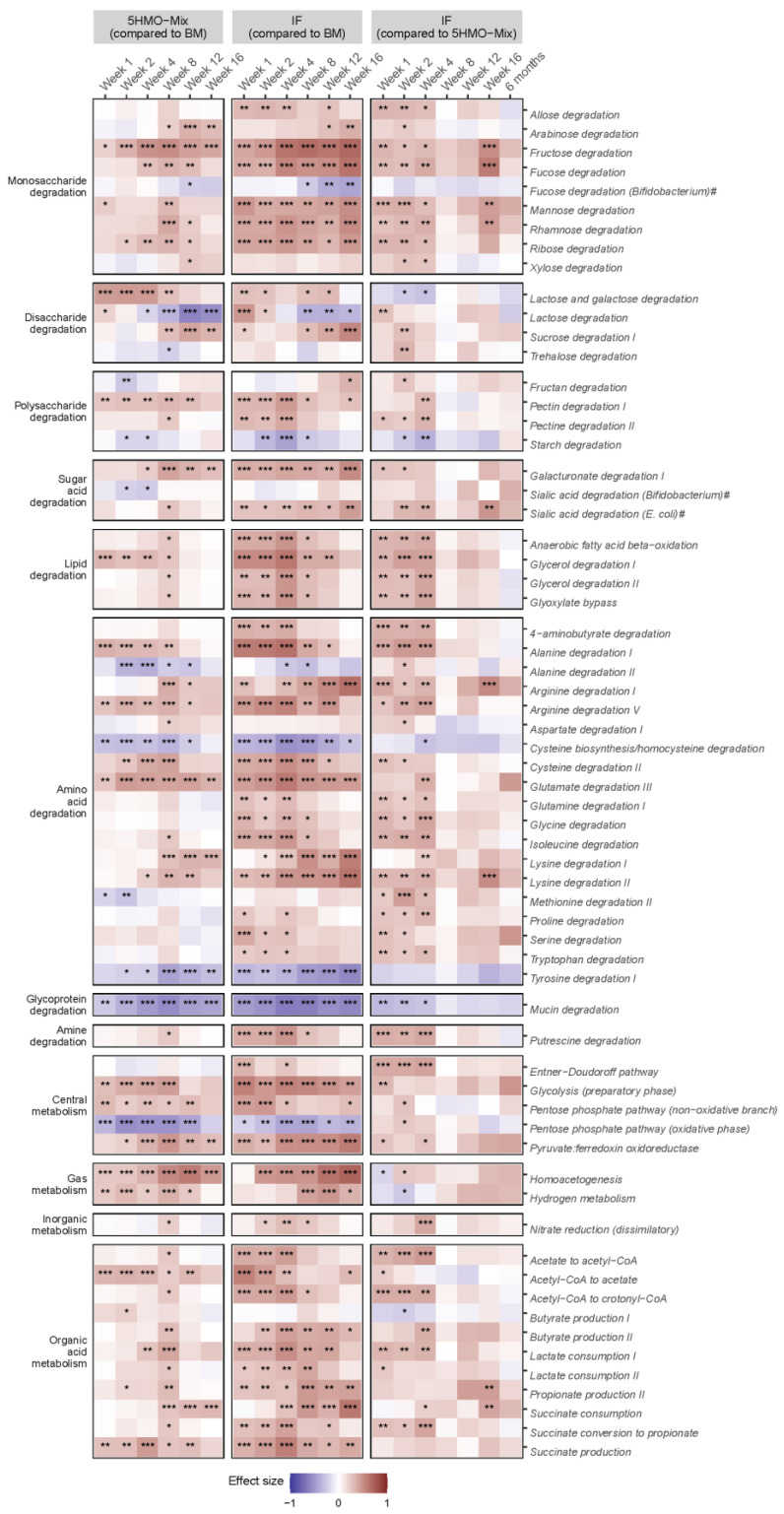
Heat map of pairwise comparisons of gut metabolic modules (GMMs) among feeding groups at each time point. Contrasts are based on the calculated effect size. The color depicts the abundance of the GMM in the group relative to the abundance in the comparator group. A positive effect size (red) shows that the GMM is more abundant relative to the comparator group, whereas a negative effect size (blue) shows the opposite. The contrasts at 6 months are shown only for the two formula groups due to the small sample size in the breastfed group (N = 2). GMMs were derived from a catalog [35] or manually annotated based on species-dependent pathways (indicated by a hash sign). Significance (*p*-values passing multiple testing): * *p* < 0.05, ** *p* < 0.01, *** *p* < 0.001.

We described the metabolic capabilities of the microbiome as Gut Metabolic Modules (GMMs) [35]. This method can be used to describe functional clades of microbiomes in the gut environment and to compare the relative abundance of specific metabolic pathways. Pairwise comparisons of GMMs among the three feeding groups showed that the breastfed group differed from the two formula groups, including a significant relative enrichment at all time points in GMMs related to saccharolytic fermentation, such as the pentose-phosphate pathway (oxidative phase) (*p* < 0.05) and cysteine biosynthesis/homocysteine degradation (*p* < 0.05). Interestingly, the module for *Bifidobacterium*-specific fucose degradation was significantly enriched in the breastfed group compared with the 5HMO-mix group in Week 12 (*p* < 0.05) and the IF control group in Weeks 8–16 (*p* < 0.05) (Figure 5). However, the two formula groups also showed differences. Several GMMs related to lipid, amino acid and monosaccharide degradation were enriched in the IF control group compared with the breastfed and 5HMO-mix groups. Conversely, metabolic modules for mucin, starch and tyrosine degradation were enriched in the breastfed and 5HMO-mix groups compared with the IF control group (Figure 5).

**Figure 6:**
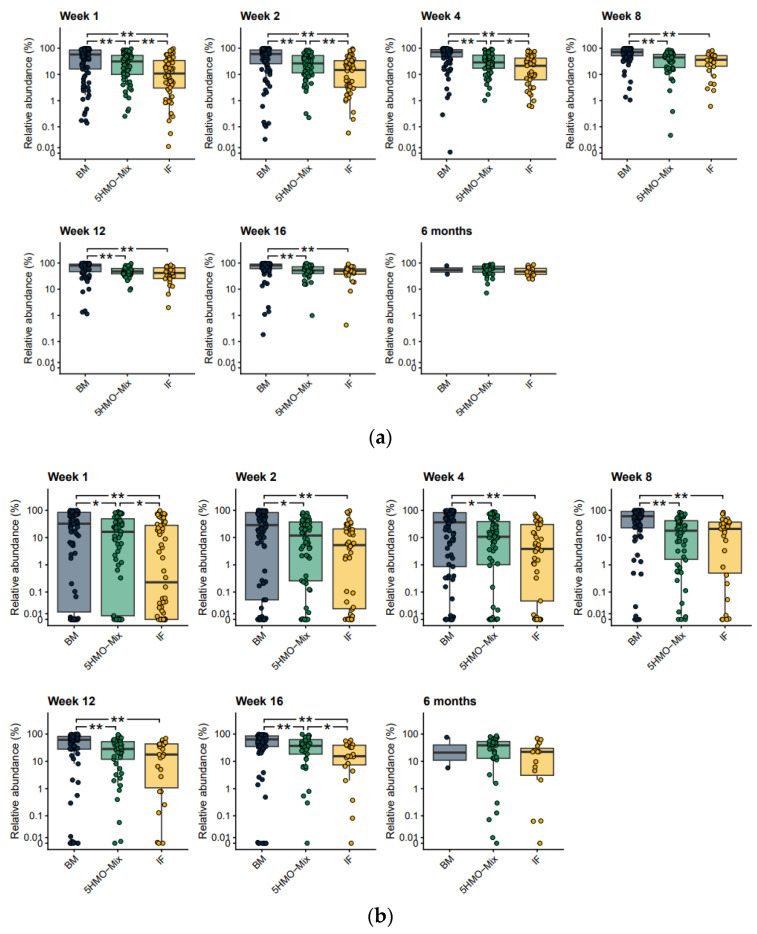
Pairwise comparisons among feeding groups at each time point for mucin and tyrosine degradation. Boxplots show the relative abundance of the summed MGS that contain the functional potential to degrade (**a**) mucins and (**b**) tyrosine in the three feeding groups at each time point. Medians are shown as horizontal lines; box boundaries indicate the interquartile range; and whiskers represent values within 1.5× the interquartile range of the first and third quartiles. Significance in pairwise comparisons was calculated using the Mann–Whitney U test (* *p* < 0.05, ** *p* < 0.01). BM = breastmilk; 5HMO-mix; IF = control infant formula. Numbers in each cohort (N) are provided in Table 1.

Specifically, we compared mucin degradation and tyrosine degradation among groups at each time point (Figure 6). Bacterial mucin degradation is a symbiotic trait [43], and tyrosine is one of the aromatic amino acids that can be converted into aromatic lactic acids by bifidobacteria [18]. We observed a significant enrichment in mucin degradation in the breastfed group compared with both formula groups at all time points (*p* < 0.01), whereas the 5HMO-mix group was significantly enriched compared with the IF group in Weeks 1–4 (*p* < 0.05) (Figure 6a). Tyrosine degradation was also significantly enriched in the breastfed group compared with both formula groups (*p* < 0.05), but the 5HMO-mix group was significantly enriched compared with the IF control group in Week 1 and Week 16 (*p* < 0.05) (Figure 6b).

## 4. Discussion

In this clinical study of healthy infants, we confirmed that breastfeeding modulates the fecal microbiome, increasing the relative abundance of bifidobacteria in the first months of life. We also found that formula supplemented with a blend of five HMOs, representing the three structural HMO types at natural concentrations, influences the fecal microbiome by shifting the structure of the community towards that of breastfed infants. 5HMO-mix affected the microbial composition after only 1 week, and these early differences persisted beyond Week 16. Although the early-life microbiome fluctuates in composition, stability and total bacterial abundance [14,16,44,45,46,47], the effect of 5HMO-mix was pronounced throughout the intervention period. Interestingly, we observed a tendency towards more significant differences between the 5HMO-mix and IF control groups at the early time points (Weeks 1–2) and again in Week 16, suggesting that supplementation ≤ 14 days after birth is directional for the effects later observed.

HMO supplementation clearly promotes the preferential growth of bifidobacteria. Importantly, the main effect of 5HMO-mix was to increase the relative abundance of *Bifidobacterium*, an effect, we showed, that was mainly due to ALDH^+^ *Bifidobacterium* associated with breastfeeding [18]. The ability of bifidobacteria to produce aromatic lactic acids through the conversion of aromatic amino acids using the ALDH enzyme is interesting because aromatic lactic acids influence the early development of the immune system [18,48]. This interaction is thought to promote intestinal homeostasis and to regulate pro-inflammatory responses, a symbiotic relationship that could be supported by providing infants with 5HMO-mix supplements. Accordingly, the metabolic module “tyrosine degradation” was enriched in the breastfed and 5HMO-mix groups compared with the IF controls, and tyrosine is one of the aromatic amino acids that can be converted into aromatic lactic acids by bifidobacteria. In infant feces, tyrosine is negatively correlated with breastfeeding and the abundance of *Bifidobacterium* [18,27,49]. To support these findings, the next step is to analyze the fecal metabolic profile. In the absence of such data, we can only speculate that the higher relative abundance of ALDH^+^ *Bifidobacterium* and increased metabolic capability for tyrosine degradation lead to higher amounts of aromatic lactic acids. In addition to these results, previous studies have shown that HMO supplements change other infant fecal metabolites, such as the short-chain fatty acid profile, which is attributed to the preferential growth of *Bacteroides* and *Bifidobacterium* at the expense of proteobacteria [50,51].

The 5HMO-mix supplements had a small effect on the *Bifidobacterium* species profile. The microbiota of breastfed infants is generally dominated by bifidobacteria that efficiently utilize HMOs, including *B. longum* subsp. *longum*, *B. bifidum*, *B. breve* and *B. longum* subsp. *infantis* [15,17,18]. However, studies also show that some breastfed infants have low abundance of bifidobacteria, and these healthy infants can be dominated by species like *Bacteroides vulgatus* and *E. coli* [15]. We found that the relative abundance of *B. longum* subsp. *longum* increased in the 5HMO-mix group compared with IF controls in the first few weeks. Other studies have shown similar modest but significant increases in the abundance of *B. longum* subsp. *infantis* in infants fed formula supplemented with two or five HMOs, respectively, when compared with control formula [24,27]. *B. longum* subsp. *infantis* is an efficient HMO utilizer, whereas *B. longum* subsp. *longum* has a limited ability to metabolize HMOs and mainly utilizes LNT [17]. Interestingly, both species are often prevalent in the gut of breastfed infants and bifidobacteria can form stable communities with the domination of a single species, or a few or multiple species [17,52]. How the 5HMO-mix supplement affects these community dynamics in the infant gut is still unclear, and further studies are needed. Nonetheless, an abundant and diverse bifidobacterial community in early life is linked to beneficial long-term health effects [53], whereas reduced abundance and diversity of bifidobacteria are associated with conditions such as atopic dermatitis [54,55], allergies [56] and childhood obesity [57]. Larger cohort studies have associated microbial composition in early life with the onset of disease later in life, indicating that host–microbiome interactions during this period are important for the development of the immune system [58,59].

The relative abundance of potential opportunistic pathogenic species was lower in the breastfed and 5HMO-mix groups than in the IF control group in the early weeks of the study. Toxicogenic *C. difficile* was previously found to be less abundant in infants receiving supplements of five HMOs compared with control formula [24], and the prevalence of fucose pathways associated with *E. coli* decreased in infants receiving formula supplemented with 2′-FL compared with control formula [23]. We also observed a significant decrease in the relative abundance of *C. difficile* and *E. coli* in the 5HMO-mix group compared with the IF control group. Other studies reported lower incidence of respiratory tract infections and reduced need for antibiotics in infants receiving formula supplemented with a mix of 2′-FL and LNnT compared with control formula [6,27]. Nevertheless, none of the species we identified were associated with toxigenicity; all enrolled infants were healthy, and adverse events were equivalent in the three feeding groups during the 6 months of intervention. Breastfed infants are better protected against bacterial infections than formula-fed infants, and the lower abundance of potential opportunistic pathogens in infants receiving HMO supplements may indicate a similar beneficial effect [49,60,61,62,63].

We investigated the metabolic capabilities of the microbiome, revealing additional features shared by breastfed infants and those receiving 5HMO-mix supplements. Metabolic modules related to amino acid and lipid degradation were significantly enriched in the IF control group compared with the 5HMO-mix and breastfed groups. Other studies have reported that protein metabolism fueled by amino acid degradation is more prevalent in formula-fed compared with breastfed infants [49,62]. This may reflect the higher protein content of the formula product compared with breastmilk and the resultant altered bacterial community [61,64]. However, the 5HMO-mix formula and the control formula used in the current study were matched 1:1 in terms of protein content [26]. Interestingly, fermentation experiments with fecal microbes showed that the protein content correlated positively with the abundance of *Escherichia* and enrichment in metabolic modules for amino acid degradation [65]. In the adult gut, the fermentation of amino acids produces metabolites that are undisputedly detrimental to health, but the implications for infant health remain unknown [66,67].

The metabolic module “mucin degradation” was significantly enriched in the breastfed group in particular but also in the 5HMO-mix group compared with the IF control group. The metabolism of mucins by gut bacteria is considered a health marker, and mucin-degrading species such as *Akkermansia muciniphila*, *Bacteroides* spp. and *B. bifidum* are identified as human gut symbionts [43,68,69,70]. Interestingly, other studies with HMO supplements showed an increase in the relative abundance of *Akkermansia* and *Bacteroides* when compared with baseline or control groups [51,71,72]. Species of *Bacteroides* are known HMO utilizers [73], while the link to *Akkermansia* is more unclear but could be associated with cross-feeding mechanisms in the gut [74]. However, to our knowledge, this is the first study linking HMO supplements with enhanced mucin degradation by the infant gut microbiome. This could be important because it is suggested that mucin glycans provide nutrients for beneficial bacteria when breastfeeding commences, which in turn allows a stable bacterial community to develop [75].

## 5. Conclusions

We have shown that infant formula supplemented with 5HMO-mix at natural concentrations supports the development of the early-life microbiome and shifts its composition closer to that of breastfed infants compared with infants receiving control formula. We confirmed that HMO supplements increase the abundance of fecal bifidobacteria, particularly ALDH^+^ bifidobacteria associated with breastfeeding. Furthermore, the analysis of microbial metabolic capability revealed functional changes, including the enrichment of tyrosine and mucin degradation. These findings could support future studies in unraveling more causal links among the infant microbiome, immune system development and long-term health effects.

## Figures and Tables

**Table 1:** Study design showing the number (N) of fecal samples analyzed at the different sample time points within each feeding group.

Sample Point	Breastmilk (BM) Group	5HMO-mix Supplement Group	Infant Formula (IF) Control Group
Week 1	90	65	56
Week 2	89	66	46
Week 4	85	59	41
Week 8	79	54	30
Week 12	81	49	26
Week 16	80	41	23
Month 6	2	33	15

## Data Availability

The data presented in this study are openly available from The European Nucleotide Archive with accession number PRJEB62690.

## Supplementary Materials

The following supporting information can be downloaded at: https://www.mdpi.com/article/10.3390/nu15143087/s1, Figure S1: Relative abundance boxplots of the most abundant genera, Figure S2: Relative abundance boxplots of the most abundant *Bifidobacterium* spp. at each time point compared among feeding groups and plotted on a pseudo-logarithmic scale, Figure S3: Relative abundance boxplots of opportunistic pathogenic MGS at each time point compared among feeding groups and plotted on a pseudo-logarithmic scale to show values spanning several orders of magnitude, Table S1: Baseline characteristics of the total study cohort, Table S2: Principal coordinates analysis based on weighted UniFrac distance, Table S3: Performance (root mean squared error (RMSE), mean absolute error (MAE) and R-squared (R^2^)) of machine learning using MGS as predictive of the clinical outcomes.
