# Supplementary material for: Infant Formula Supplemented with Five Human Milk Oligosaccharides Shifts the Fecal Microbiome of Formula-Fed Infants Closer to That of Breastfed Infants
Source: Nutrients. 2023 Jul 10;15(14):3087. doi: 10.3390/nu15143087 (PMC10383262; doi:10.3390/nu15143087)
Supplement: Supplementary file 1 [file nutrients-15-03087-s001.zip › supplementary information nutrients-2474467.pdf]

Table S1. Baseline characteristics of the total study cohort (safety set, n = 311).

	**5HMO-Mix n = 103**	**IF n = 104**	**BM n = 104**	**Total n = 311**
Sex, *n* (%)				
Male	54 (52.4)	55 (52.9)	52 (50.0)	161 (51.8)
Female	49 (47.6)	49 (47.1)	52 (50.0)	150 (48.2)
Age at enrollment, days	4.8±3.2 (1-16)	4.3±2.7 (1-14)	5.2±3.6 (1-14)	4.8±3.2 (1-16)
Gestational age at birth, weeks	39.3±1.1	39.3±1.2	39.4±1.1	39.3±1.1
Ethnicity, *n* (%)				
Caucasian	95 (92.2)	100 (96.2)	97 (93.3)	292 (93.9)
African	1 (1.0)	1 (1.0)	-	2 (0.6)
Mixed	6 (5.8)	3 (2.9)	4 (3.8)	13 (4.2)
Other	1 (1.0)	-	3 (2.9)	4 (1.3)
Mode of delivery, *n* (%)				
Vaginal	56 (54.1)	64 (61.5)	73 (70.2)	193 62.1)
Cesarean section	32 (31.1)	33 (31.7)	24 (23.1)	
Assisted vaginal	15 (14.6)	7 (6.7)	7 (6.7)	29 (9.3)
Birth weight, g	3321.8±434.7	3351.1±405.5	3412.0±381.3	3361.8±408.1
Birth length, cm	50.04±2.01	50.44±2.23	50.54±1.97	50.34±20.8
Birth head circumference, cm	34.65±1.13	34.68±1.23	34.65±1.48	34.66±1.29
APGAR score[1]	10.0±0.2 (9-10)	9.9±0.3 (9-10)	9.9±0.3 (9-10)	9.9±0.3 (9-10)
Use of antibiotics by mother before or during delivery, *n* (%)	15 (14.6)	20 (19.2)	22 (21.2)	
Feeding history from birth up to enrollment, *n* (%)				
Breast milk fed	11 (10.7)	6 (5.8)	93 (92.3)	
Infant formula fed	92 (89.3)	98 (94.2)	8 (7.7)	

[1] Mean±SD (min-max)

Table S2. Principal Coordinates Analysis (PCoA) based on weighted UniFrac distance. P-values for permutational multivariate analysis of variance (PERMANOVA) and R^2 for effect sizes.

	**All groups**		**BM vs 5HMO-mix**		**BM vs IF control**		**5HMO-mix vs IF control**	
	R^2	**P**	R^2	**P**	R^2	**P**	R^2	**P**
Week 1	8.48%	0.001	4.72%	0.001	10.23%	0.001	2.98%	0.013
Week 2	10.53%	0.001	7.32%	0.001	10.8%	0.001	3.89%	0.004
Week 4	12.75%	0.001	8.5%	0.001	13.88%	0.001	4.08%	0.008
Week 8	11.98%	0.001	9.39%	0.001	12.79%	0.001	1.71%	0.179
Week 12	9.02%	0.001	5.59%	0.003	9.52%	0.001	3.86%	0.031
Week 16	10.70%	0.001	3.34%	0.012	13.32%	0.001	8.06%	0.002
6 months	NA	NA	NA	NA	NA	NA	4.86%	0.072

Table S3. Performance (root mean squared error [RMSE], mean absolute error [MAE], and R-squared [R2]) of the machine learning using MGS as predictive of the selected clinical outcomes.

**Variable**	**RMSE**	**MAE**	**R^2**
Spitting up	0.693	0.517	-0.036
Vomiting	0.232	0.17	0.038
Flatulence / day	0.56	0.402	-0.037
Fussiness without crying / day	0.537	0.477	-0.041
Crying / day	0.492	0.409	-0.003
Awaking at night/day	0.545	0.466	0.002
Watery stool/day	1.298	1.044	0
Watery stool/visit	2.645	1.499	0.02
Soft stool/visit	5.128	4.107	0.142
Soft stool/day	1.465	1.138	0.182
Movements/visit	1.418	1.123	0.326
Movements/day	1.425	1.134	0.316
Movements	1.357	1.071	0.261
Hard stools/visit	0.801	0.302	0.003
Hard stools/day	0.696	0.588	-0.163
Formed stool/visit	3.044	1.98	0.112
Formed stool/day	1.224	0.899	0.076

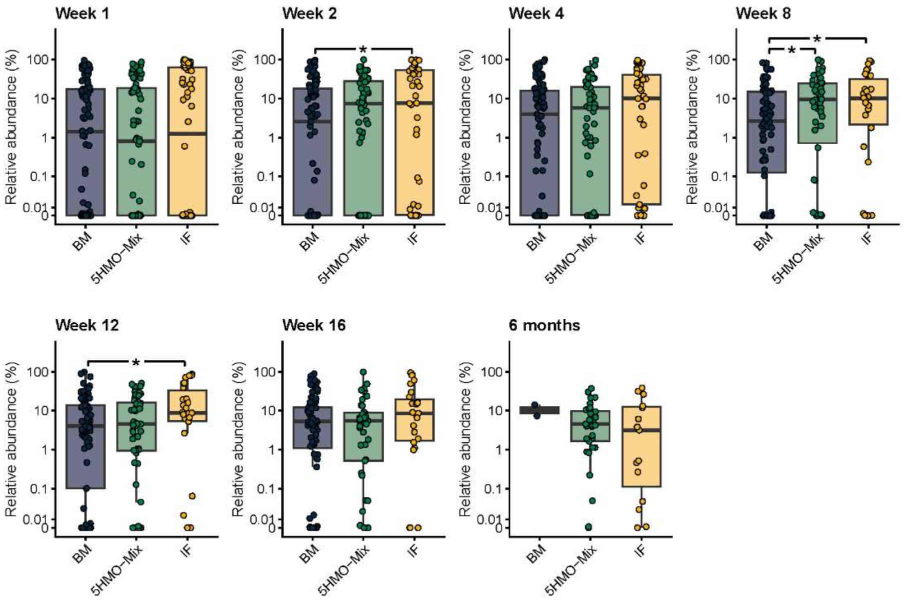
Week 1
Week 2
Week 4
Week 8
Week 12
Week 16
6 months
Relative abundance (%)
BM
5HMO−Mix
IF

(a)

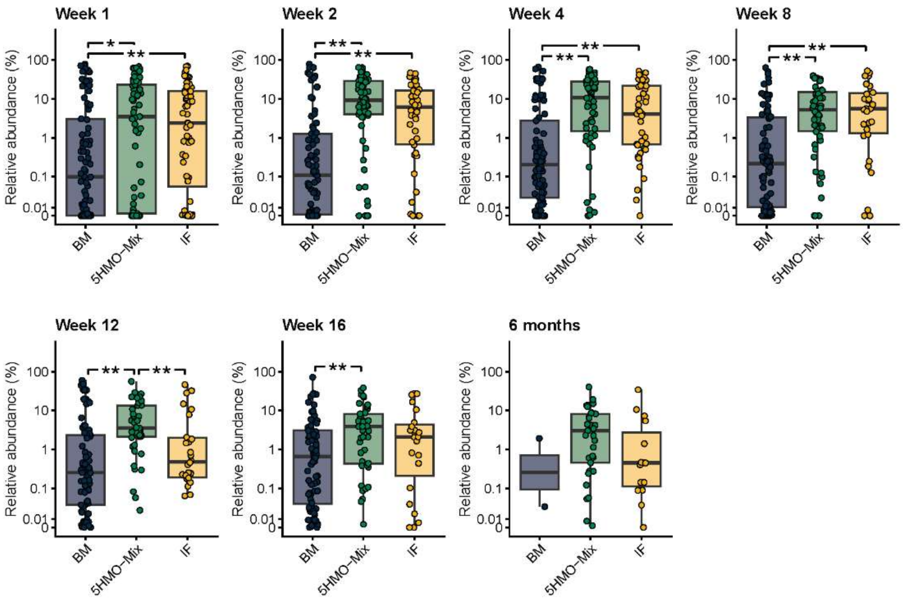
Week 1
Week 2
Week 4
Week 8
Week 12
Week 16
6 months
Relative abundance (%)
BM
5HMO−Mix
IF

(b)

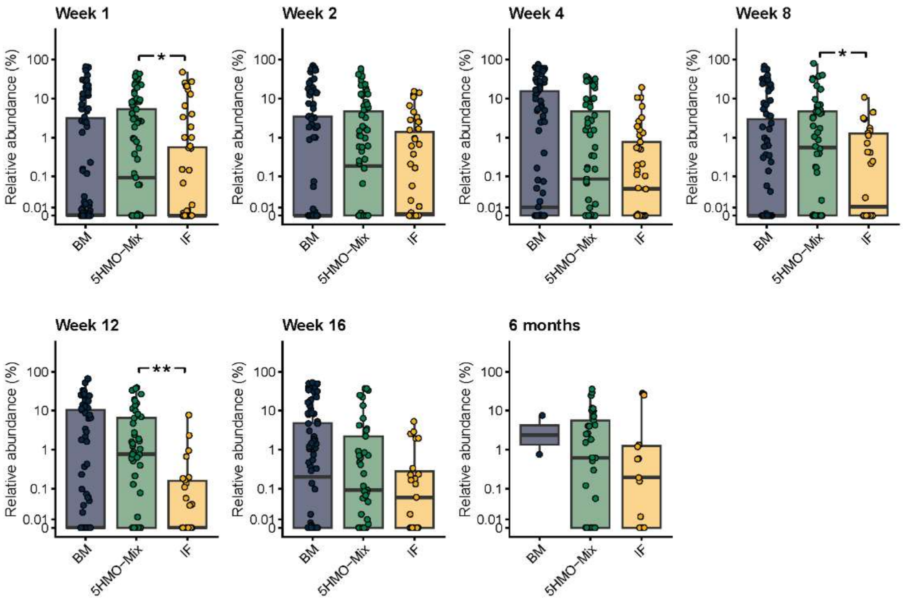
Week 1
Week 2
Week 4
Week 8
Week 12
Week 16
6 months
Relative abundance (%)
BM
5HMO−Mix
IF

(c)

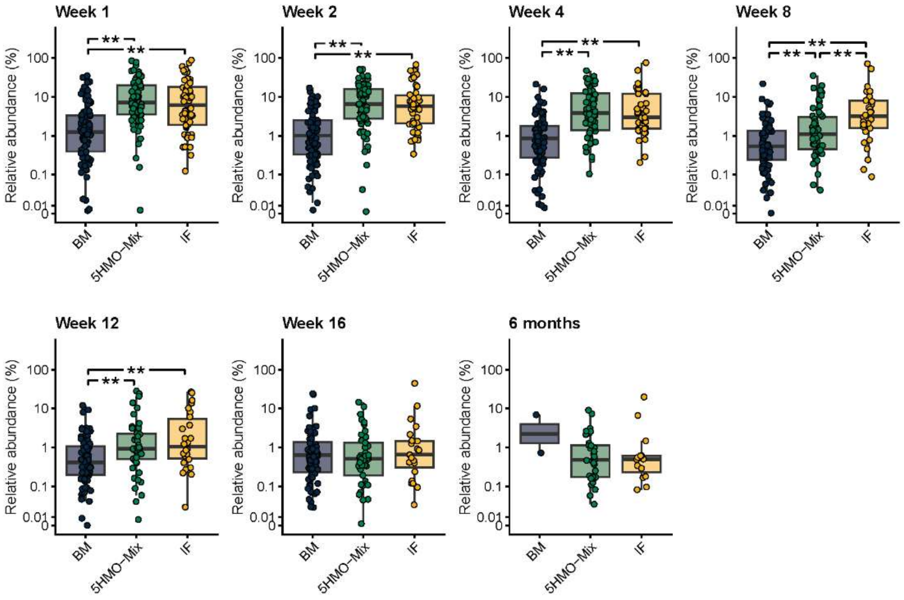
Week 1
Week 2
Week 4
Week 8
Week 12
Week 16
6 months
Relative abundance (%)
BM
5HMO−Mix
IF

(d)

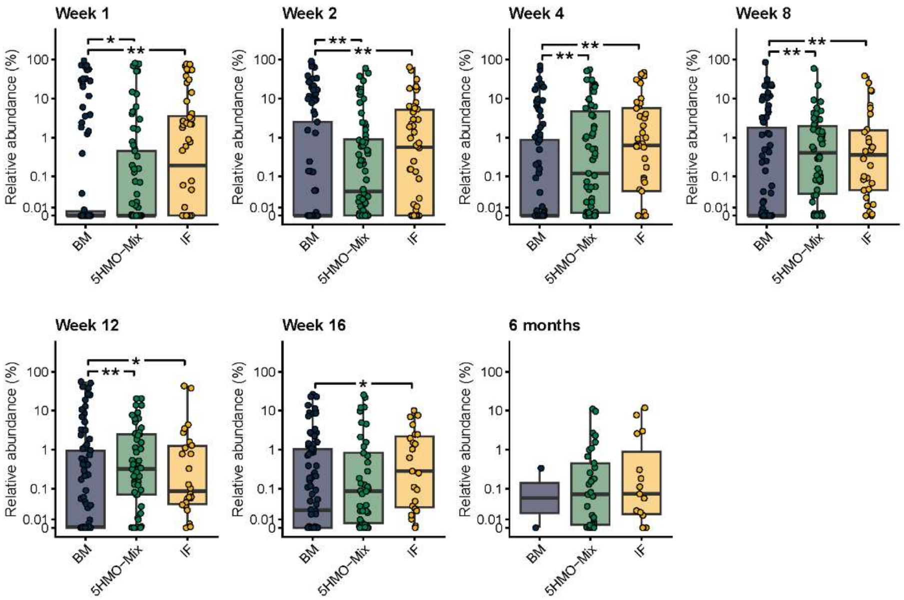
Week 1
Week 2
Week 4
Week 8
Week 12
Week 16
6 months
Relative abundance (%)
BM
5HMO−Mix
IF

(e)

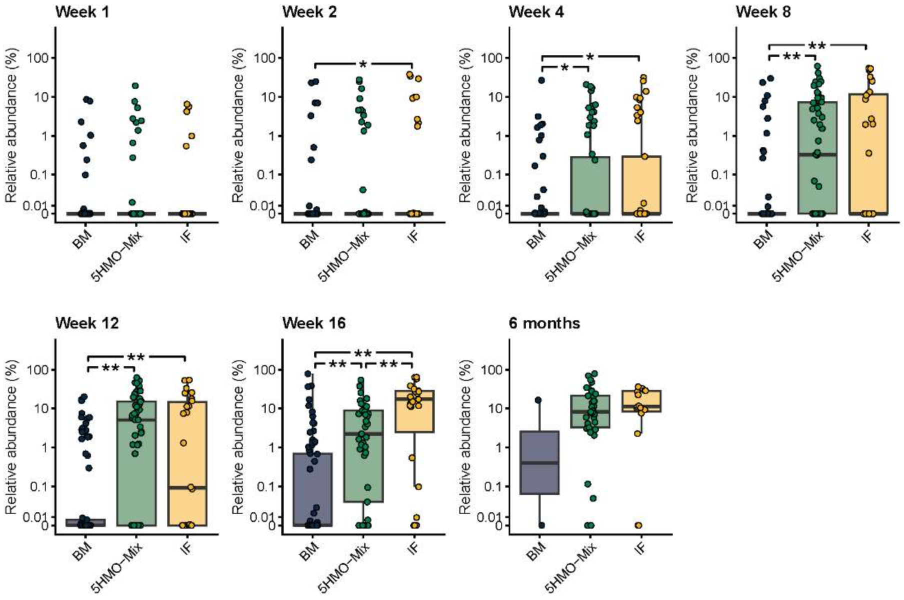
Week 1
Week 2
Week 4
Week 8
Week 12
Week 16
6 months
Relative abundance (%)
BM
5HMO−Mix
IF

(f)

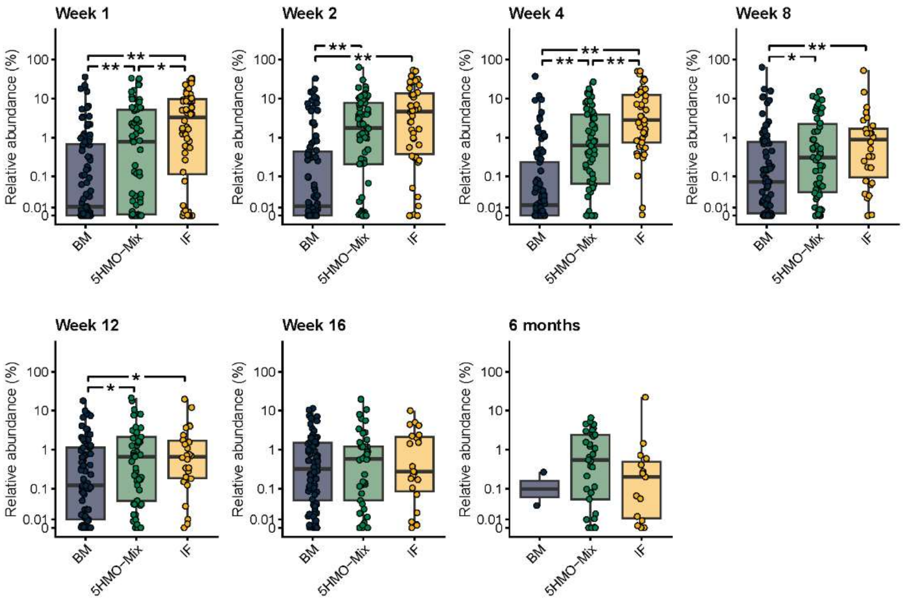

(g)

Supplementary Figure S1

Relative abundance boxplots of the most abundant (on average >85% of the analyzed MGS) genera (A) *Escherichia*, (B) *Veillonella*, (C) *Bacteroides*, (D) *Streptococcus*, (E) *Klebsiella*, (F) *Blautia*, (G) *Enterococcus* at each time point compared between feeding groups. Boxplots show the median as horizontal lines; box boundaries indicate the interquartile range; whiskers represent values within 1.5 × the interquartile range of the first and third quartiles. MGS are identified at genus level. Significance in pairwise comparison was calculated using Mann-Whitney U-test (*p < 0.05, **p < 0.01. BM = breastmilk, 5HMO-mix, IF = control infant formula. Numbers in each cohort (N) are provided in Table 1

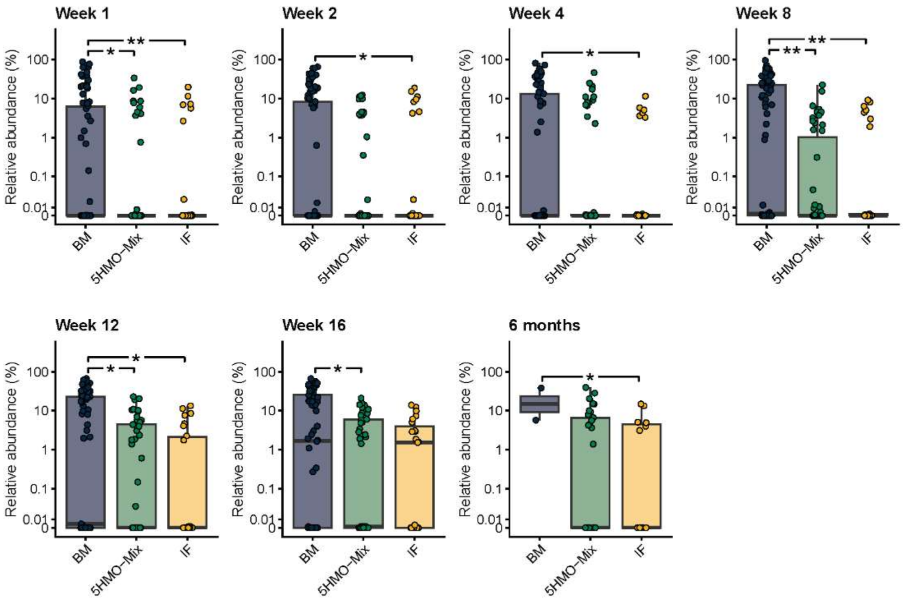
Week 1
Week 2
Week 4
Week 8
Week 12
Week 16
6 months
Relative abundance (%)
BM
5HMO−Mix
IF

(a)

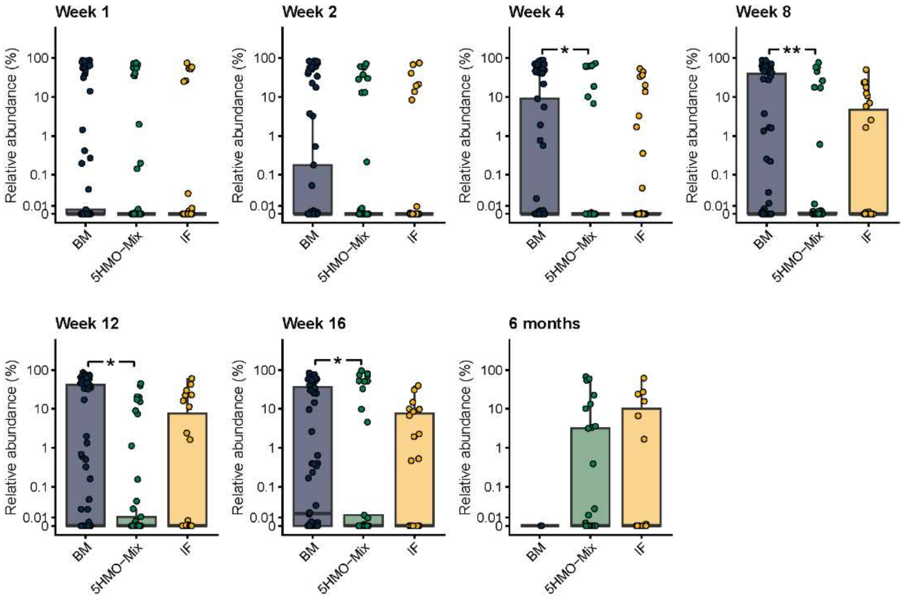
Week 1
Week 2
Week 4
Week 8
Week 12
Week 16
6 months
Relative abundance (%)
BM
5HMO−Mix
IF

(b)

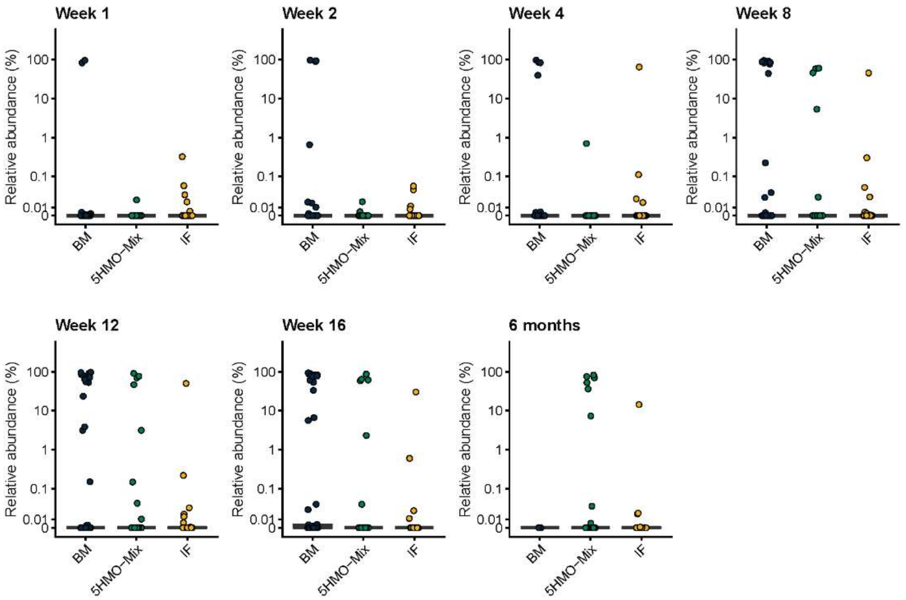
Week 1
Week 2
Week 4
Week 8
Week 12
Week 16
6 months
Relative abundance (%)
100
10
1
0.1
0.01
0
BM
5HMO−Mix
IF

(c)

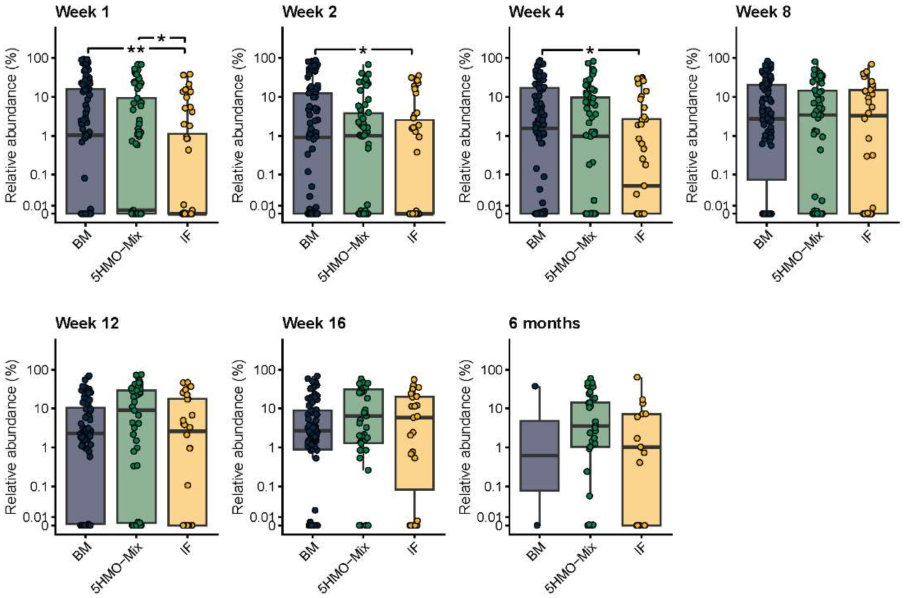
Week 1
Week 2
Week 4
Week 8
Week 12
Week 16
6 months
Relative abundance (%)
100
10
1
0.1
0.01
0
BM
5HMO−Mix
IF
**
*

(d)

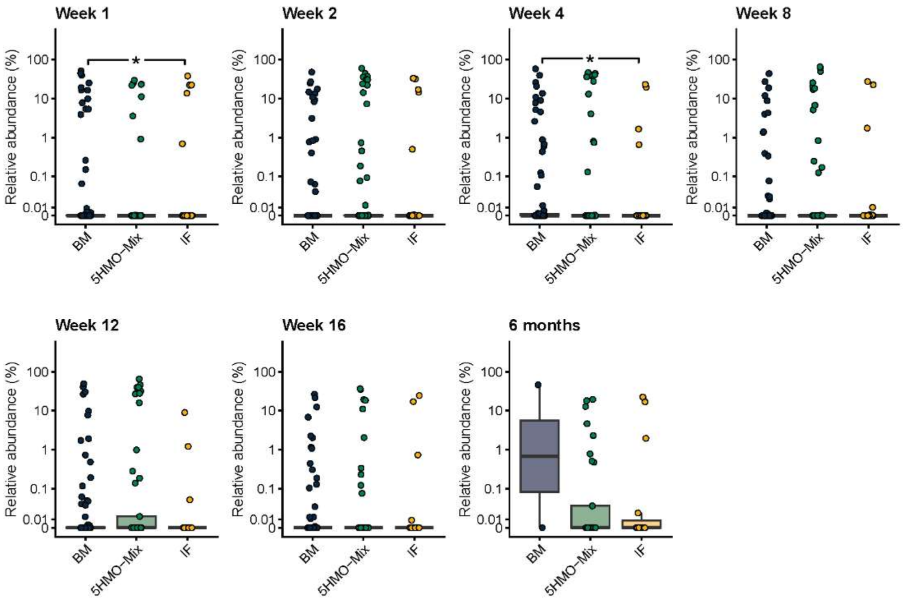
Week 1
Week 2
Week 4
Week 8
Week 12
Week 16
6 months
Relative abundance (%)
100
10
1
0.1
0.01
0
*
BM
5HMO−Mix
IF

(e)

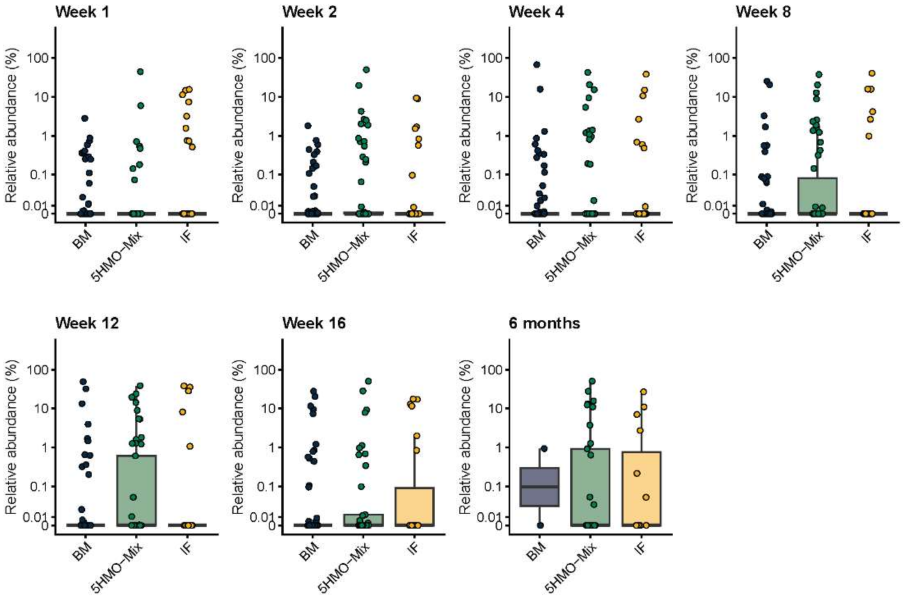
Week 1
Week 2
Week 4
Week 8
Week 12
Week 16
6 months
Relative abundance (%)
100
10
1
0.1
0.01
0
BM
5HMO−Mix
IF

(f)

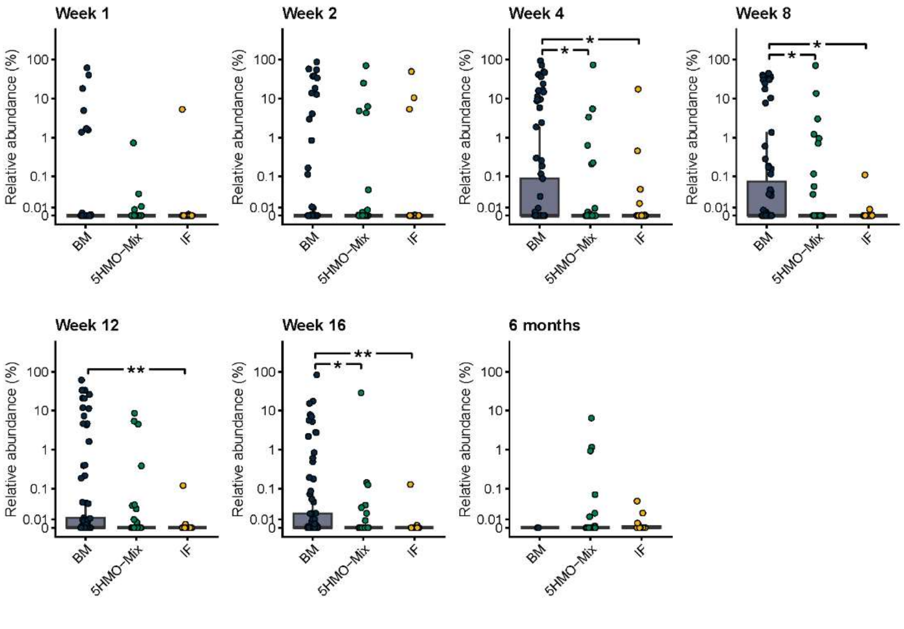
Week 1
Week 2
Week 4
Week 8
Week 12
Week 16
6 months
Relative abundance (%)
100
10
1
0.1
0.01
0
BM
5HMO−Mix
IF
*
**

(g)

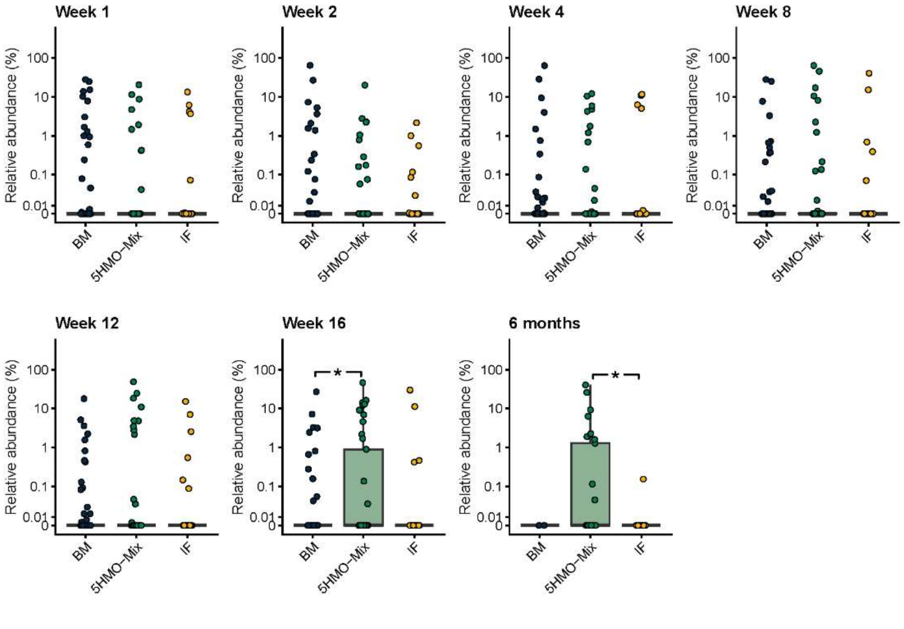
Week 1
Week 2
Week 4
Week 8
Week 12
Week 16
6 months
Relative abundance (%)
100
10
1
0.1
0.01
0
BM
5HMO−Mix
IF
*

(h)

Supplementary Figure S2

Relative abundance boxplots of the most abundant (on average across groups and time points >10% MGS) Bifidobacterium spp. at each time point compared between feeding groups and plotted on a pseudo-logarithmic scale. (A) *B. bifidum*, (B) *B. breve*, (C) *B. longum subsp. Infantis*, (D) *B. longum subsp. longum*, (E) B. *pseudocatenulatum*, (F) *B. catenulatum subsp. kashiwanohense*, (G) *B. dentium* and (H) *B. adolescentis*. Boxplots show the median as horizontal lines; box boundaries indicate the interquartile range; whiskers represent values within 1.5 × the interquartile range of the first and third quartiles. MGS are identified at species or subspecies level. Significance in pairwise comparison was calculated using Mann-Whitney U-test (*$p < 0.05$, **$p < 0.01$. BM = breastmilk, 5HMO-mix, IF = control infant formula. Numbers in each cohort (N) are provided in Table 1.

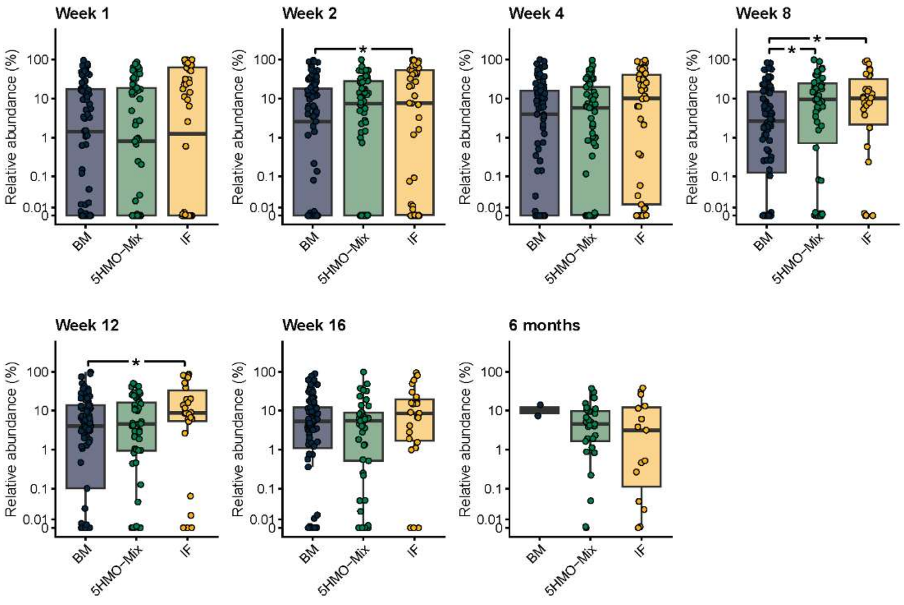
Week 1
Week 2
Week 4
Week 8
Week 12
Week 16
6 months
Relative abundance (%)
BM
5HMO−Mix
IF

(a)

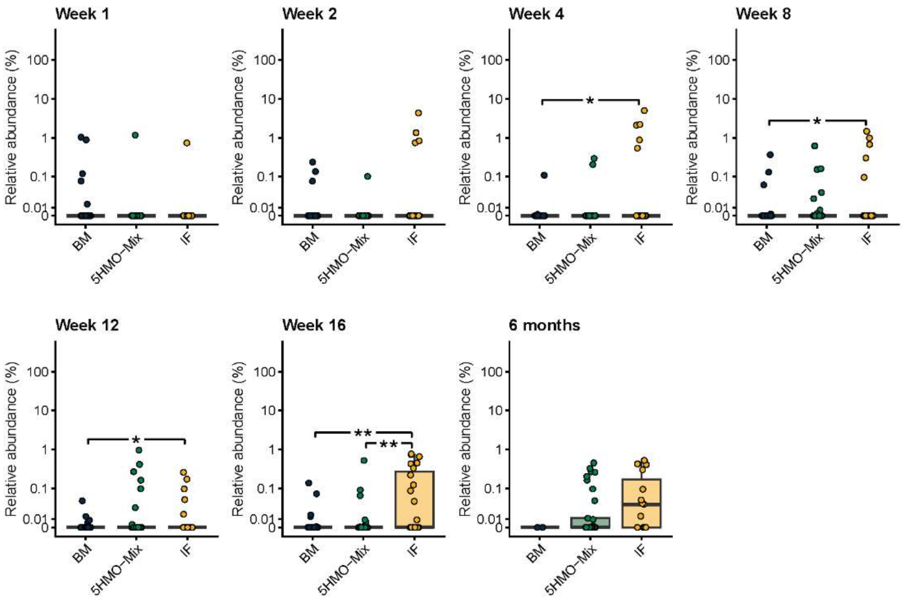
Week 1
Week 2
Week 4
Week 8
Week 12
Week 16
6 months
Relative abundance (%)
BM
5HMO−Mix
IF

(b)

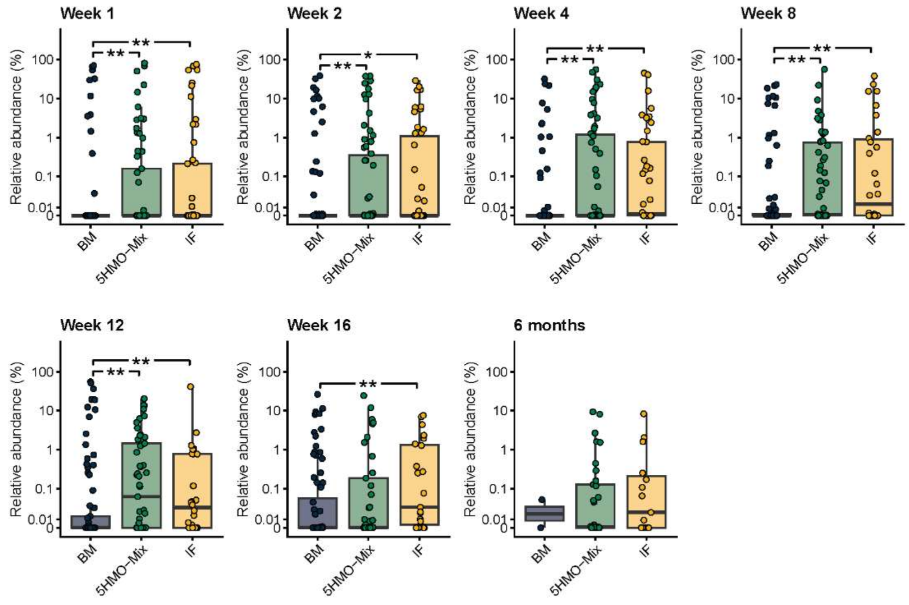

(c)

<u>Supplementary Figure S3</u>

Relative abundance boxplots of opportunistic pathogenic MGS at each timepoint compared between feeding groups and plotted on a pseudo-logarithmic scale to show values spanning several orders of magnitude. Boxplots show the median as horizontal lines; box boundaries indicate the interquartile range; whiskers represent values within 1.5 × the interquartile range of the first and third quartiles. MGS are identified at species level as (A) *Escherichia coli*, (B) *Clostridioides difficile* and (C) *Klebsiella pneumonia*. Significance in pairwise comparison was calculated using Mann-Whitney U-test (*p < 0.05, **p < 0.01. BM = breastmilk, 5HMO-mix, IF = control infant formula. Numbers in each cohort (N) are provided in Table 1
